# Oncogene Activation Induces Metabolic Transformation Resulting in Insulin-Independence in Human Breast Cancer Cells

**DOI:** 10.1371/journal.pone.0017959

**Published:** 2011-03-17

**Authors:** Aliccia Bollig-Fischer, T. Gregory Dewey, Stephen P. Ethier

**Affiliations:** 1 Department of Oncology, Wayne State University School of Medicine, Detroit, Michigan, United States of America; 2 Breast Cancer Biology Program, Karmanos Cancer Institute, Detroit, Michigan, United States of America; 3 University of La Verne, Los Angeles, California, United States of America; Florida International University, United States of America

## Abstract

Normal breast epithelial cells require insulin and EGF for growth in serum-free media. We previously demonstrated that over expression of breast cancer oncogenes transforms MCF10A cells to an insulin-independent phenotype. Additionally, most breast cancer cell lines are insulin-independent for growth. In this study, we investigated the mechanism by which oncogene over expression transforms MCF10A cells to an insulin-independent phenotype. Analysis of the effects of various concentrations of insulin and/or IGF-I on proliferation of MCF10A cells demonstrated that some of the effects of insulin were independent from those of IGF-I, suggesting that oncogene over expression drives a true insulin-independent proliferative phenotype. To test this hypothesis, we examined metabolic functions of insulin signaling in insulin-dependent and insulin-independent cells. HER2 over expression in MCF10A cells resulted in glucose uptake in the absence of insulin at a rate equal to insulin-induced glucose uptake in non-transduced cells. We found that a diverse set of oncogenes induced the same result. To gain insight into how HER2 oncogene signaling affected increased insulin-independent glucose uptake we compared HER2-regulated gene expression signatures in MCF10A and HER2 over expressing MCF10A cells by differential analysis of time series gene expression data from cells treated with a HER2 inhibitor. This analysis identified genes specifically regulated by the HER2 oncogene, including VAMP8 and PHGDH, which have known functions in glucose uptake and processing of glycolytic intermediates, respectively. Moreover, these genes specifically implicated in HER2 oncogene-driven transformation are commonly altered in human breast cancer cells. These results highlight the diversity of oncogene effects on cell regulatory pathways and the importance of oncogene-driven metabolic transformation in breast cancer.

## Introduction

Tumor cell metabolism is unlike that of normal cells. The earliest recognized metabolic distinction for cancer cells is their adaptation to metabolize glucose by glycolysis even when there is sufficient oxygen to metabolize glucose via the Krebs cycle. Known as the Warburg effect, it is a common feature of cancer cells [1]. Compared to oxidative phosphorylation, aerobic glycolysis produces less ATP per molecule of glucose, but it is advantageous for cancer growth because of the increased availability of glycolytic intermediates to produce biosynthetic precursors, including amino acids, lipids and nucleotides. In conjunction with aerobic glycolysis, increased fatty acid synthesis and mitochondrial glutamine metabolism contribute to enhanced tumor cell metabolism that provides an abundance of cellular building blocks necessary for unmitigated cell growth and proliferation [2], [3], [4]. Metabolic pathways and enzymes have been identified as important regulators of cancer cell growth [5], [6], [7], [8], and what is already known of cancer cell metabolism has been successfully exploited to image cancer in patients through detection of enhanced uptake of ^18^F-deoxyglucose by positron emission tomography (FDG-PET) [9]. Recently, metabolic targeting has emerged as a therapeutic strategy resulting in novel types of anticancer agents that could have broad therapeutic applications [10].

Recent studies suggest that proto-oncogenes exert regulatory effects on metabolism in normal cells, and that tumorigenic mutations and genomic amplification of these genes contribute to the metabolic autonomy observed in tumor cells [7]. Thus, oncogene signaling not only activates cancer cell mitogenic pathways that drive unchecked growth of cancer, but also promotes a coordinated metabolic transformation of cancer cells by activating metabolic pathways and transcriptionally regulating metabolic enzymes. PI3K/AKT, Ras, cMyc and HER2 are examples of oncogenes that promote growth factor-independent growth and metabolic autonomy in cancer cells [11], [12], [13], [14]. In breast cancer cells, the HER2 oncogene activates signaling pathways that regulate the activities of PI3K/AKT, Ras, mTOR, Src, and HIF1α [15], [16], [17]. Also, HER2 signaling has been shown to transcriptionally up-regulate the glycolytic enzyme LDHA [12]. Reports describing cancer cell metabolism are abundant, but aerobic glycolysis and many of the same metabolic pathways and metabolic enzymes activated in cancer cells are also up-regulated or activated in rapidly proliferating normal cells [5], [18], [19], [20], [21]. If an anticancer strategy through metabolic molecular targeting is to be realized it must be determined how cancer cell metabolism is abnormally regulated compared to normal proliferating cells. But, how the metabolism of normal proliferating cells and cancer cells differ has not yet been completely elucidated.

In several studies from our laboratory, we have demonstrated that oncogenes can transform normal cells to a state of insulin-independence [22], [23]. For many years, the acquisition of insulin-independence was considered to be a surrogate for IGF-I independence and was thus associated with the mitogenic effects of the oncogenes. However, in the present study, we show that oncogene-induced insulin-independence also represents a metabolic transformation, which is a result of oncogene-regulated changes to gene transcription. Thus, the ability to induce metabolic transformation is a key feature of the HER2 oncogene as well as other important breast cancer oncogenes,. In this report we present novel methods to analyze an oncogene-regulated transcriptome to identify dysregulated genes in a transformed cell that participate in the emergence of metabolic changes associated with insulin-independent proliferation, particularly genes related to glucose uptake and glycolysis.

## Results

**Figure 1:**
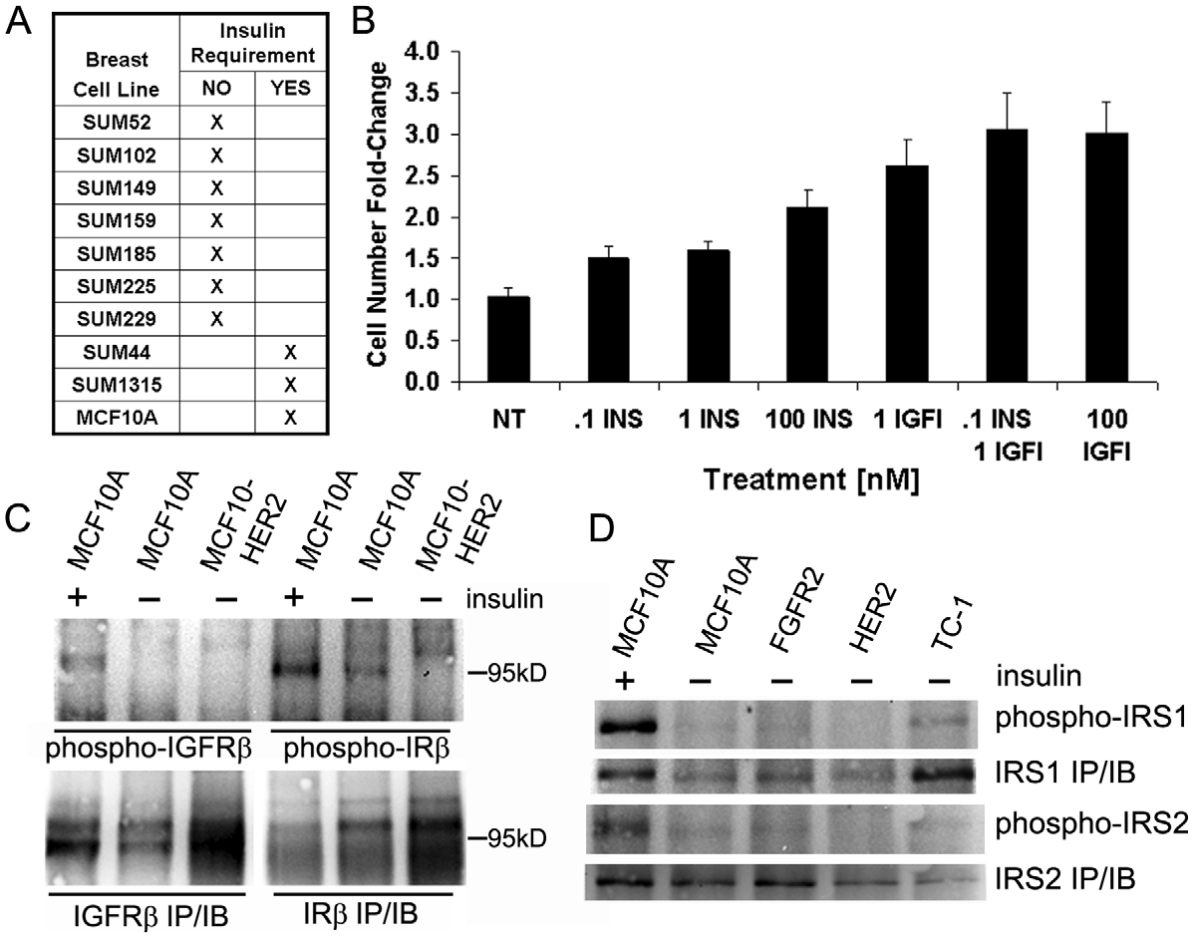
Receptor activation and insulin-induced proliferation in breast cells. (A) A list of breast cancer cell lines indicating if they proliferate without insulin in serum-free conditions, including the nontransformed breast epithelial cell line MCF10A. (B) The effects of a range of insulin and IGF-I treatments on proliferation of MCF10A cells in 72 hours. Physiological concentrations of insulin and IGF-I increased MCF10A proliferation (*Bars*, standard error). (C) Immunoblot analysis of phosphorylated IGF-IR and IR in MCF10A and MCF10HER2 cells. Receptors were immunoprecipitated from 1 mg of whole cell lysate, 50% of the eluent was loaded per gel lane and either tyrosine phosphorylated (phospho, *upper*) or total receptor (IP/IB, *lower*) levels were probed. (D) Immunoblot analysis of phosphorylated IRS1 and IRS2 in MCF10A and MCF10A-derived cell lines transduced to stably over express HER2, TC1, and FGFR2 oncogenes. Samples were immunoprecipitated from 1 mg of whole cell lysate, 50% of the eluent was loaded onto gel lanes and probed for either total IRS1 (IRS1 IP/IB) or phosphorylated (phospho-) IRS1 protein. Immunoprecipitated phosphorylated IRS2 levels (phospho) and total IRS2 (IRS2 IP/IB) were similarly detected.

Several years ago, we developed a serum-free cell culture system that supports continuous proliferation of human mammary epithelial (HME) cells. Using these culture conditions, we and others found that HME cells in general, and MCF10A cells in particular, have an absolute requirement for insulin and EGF for continuous growth in serum-free media. More recently, we have shown that MCF10A cells stably transduced to over express known oncogenes no longer require insulin for proliferation, though they still depend on EGF [22], [23]. Among the oncogenes that can transform cells to insulin-independence are HER2, WHSC1L1, TC1, DDHD2 and FGFR2. Furthermore, these oncogene over expressing, insulin-independent cells exhibit other transformed phenotypes such as anchorage-independent growth and disrupted epithelial architecture in three-dimensional morphogenesis assays [22], [23]. Of significance, we have observed the insulin-independence phenotype in most of the human breast cancer cell lines developed in our laboratory (Figure 1A).

The supraphysiological concentration of insulin used to routinely culture MCF10A cells causes activation of both the IR and IGF-IR [24]. To examine the relative contributions of signaling from the IR and the IGF-IR in MCF10A cell proliferation, we measured the proliferative response of MCF10A cells to various concentrations of insulin in our serum-free culture conditions, including low concentrations that do not bind the IGF-IR. MCF10A cells cultured without insulin showed no significant increase in cell number during the experiment. MCF10A cells treated with insulin at the physiological concentration of 0.1 nM demonstrated a 50% increase in cell number in 72 hours (Figure 1B). Treatment with a 10-fold greater concentration of insulin (1 nM) elicited no further increase in proliferation; however, insulin at 100 nM resulted in a 110% increase in cell number. IGF-I at the physiological concentration of 1 nM induced a 160% increase in MCF10A cell numbers (Figure 1B), and co-treatment with 0.1 nM insulin plus 1 nM IGF-I induced an additional 45% increase in cell number. Cell proliferation induced in co-treated cultures was equal to the response induced by a supraphysiological concentration of IGF-I (200%), which binds both the IR and IGF-IR. These results indicate that under normal growth conditions, activation of both the IR and IGF-IR plays a role in supporting cell proliferation under serum-free conditions.

To confirm the role of both receptors in the proliferative response to insulin in MCF10A cells, we immunoprecipitated IR and IGF-IR from cells treated with the standard supraphysiological concentration of insulin, and probed the immunoprecipitates for receptor abundance and for tyrosine phosphorylation. In the same experiment we examined IR and IGF-IR tyrosine phosphorylation in insulin-independent MCF10HER2 cells (MCF10A cells transformed by stable HER2 over expression). Figure 1C shows that the IR and IGF-IR were readily detectable in MCF10A cells cultured with and without insulin in serum-free media, and removal of insulin from the medium resulted in loss of IR and IGF-IR tyrosine phosphorylation (Figure 1C). In agreement with this finding, immunobloting for tyrosine phosphorylated IRS1 and IRS2, downstream targets of IR and IGF-IR kinase activity, showed that tyrosine phosphorylation of IRS1 and IRS2 in MCF10A cells was insulin-dependent (Figure 1D). In insulin-independent MCF10HER2 cells cultured in serum-free, insulin-free medium, tyrosine phosphorylated IR, IGF-IR, IRS1 and IRS2 were not detected. These results indicate that insulin-independent growth of the HER2 oncogene-transformed cell line is not the result of constitutive activation of the IR or the IGF-IR. Immunoblot analysis showed that IRS1 and IRS2 were also not tyrosine phosphorylated in insulin-independent MCF10A cells transformed by TC1 or FGFR2 cultured in serum-free media without added insulin (Figure 1D).

### Insulin regulates high-level glucose uptake by MCF10A cells, oncogene-transformed cells show high-level uptake independent of insulin

**Figure 2:**
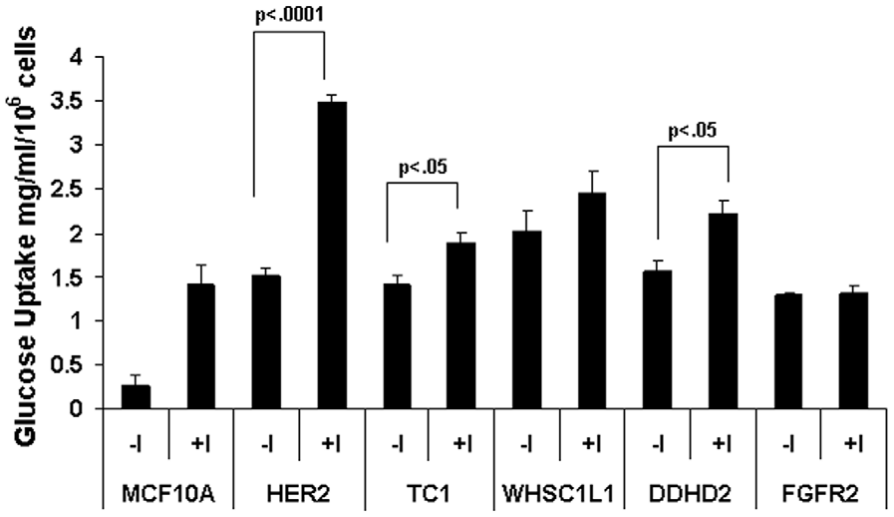
Measurement of glucose uptake by cells cultured with or without insulin. Cell lines examined were MCF10A and MCF10A transduced to over express the indicated oncogenes. Glucose uptake was determined by an enzymatic and colorometric-based absorbance assay of glucose in fresh media and in spent media after 48 hours, the difference was normalized to the number of cells in 48 hour cultures. Glucose uptake was substantially induced in MCF10A cell cultures containing insulin (+I, 5 µg/ml) compared to MCF10A minus-insulin culture conditions (-I). In contrast, in MCF10A cells transformed by oncogene over expression relatively high-level glucose uptake was observed in both insulin-containing and insulin-minus culture conditions. Relative to minus-insulin conditions glucose uptake was further significantly induced by insulin in transduced cells over expressing HER2, TC1 and DDHD2. *Bars*, standard error of three experiments.

We observed that oncogene-transformed MCF10A cells acquired independence of the mitogenic signal originating from the IGF-IR and unlike nontransformed cells no longer required insulin for proliferation in serum-free culture conditions. Next, we examined how independence from a metabolic signal originating from the IR related to glucose uptake in cultures of insulin-independent oncogene-transformed cells. In insulin-dependent cells, activated IR induces glucose uptake via facilitated transport [25]. To measure the amount of glucose taken up by insulin-dependent and insulin-independent cells, we quantified the amount of glucose in the culture media collected at the start and end of the experiment and expressed the difference relative to cell number. Measurements were taken under conditions with or without insulin added to the serum-free culture media. Results graphed in Figure 2 show that over the course of 48 hours, the amount of glucose taken up by nontransformed MCF10A cells in the absence of insulin was 0.26 mg/ml/10^6^ cells, and the presence of insulin increased glucose uptake 5.4-fold. For MCF10A cells transformed by over expression of HER2, TC1, DDHD2, WHSC1L1 or FGFR2, glucose uptake in their routine insulin-free, serum-free medium was equal to or greater than that observed for parental MCF10A cells cultured in the presence of insulin. Over expression of WHSC1L1 elicited the highest glucose uptake in the absence of insulin, 2.03 mg/ml/10^6^ cells. The addition of insulin to the media caused an additional significant increase in glucose uptake in cells over expressing HER2, TC1 or DDHD2, indicating that these cells, despite being insulin-independent, are still responsive to insulin. These data show that in nontransformed breast epithelial cells, insulin was required for high-level glucose uptake, and that oncogene transformed cells became insulin-independent for similar high-level glucose uptake. Thus, in addition to their well know effects on mitogenic signaling, oncogenes such as HER2 and others play a role in metabolic transformation.

### Status of facilitated glucose transporters in MCF10A and MCF10HER2 cells

**Figure 3:**
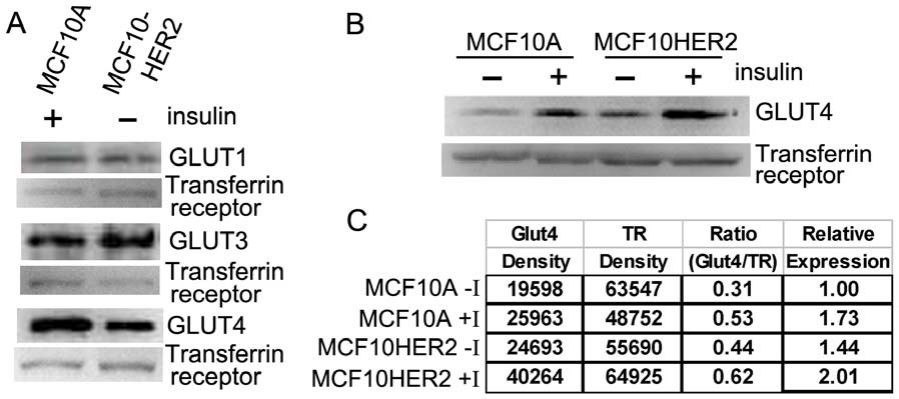
Immunoblot analysis of facilitated glucose transporters in the plasma membranes of MCF10A and MCF10HER2 cells. (A) Plasma membrane proteins were isolated from MCF10A cells cultured with insulin (5 µg/ml) and MCF10HER2 cells cultured without insulin. Isolated membrane proteins were probed for GLUT1, GLUT3 and GLUT4. Transferrin receptor was used as a plasma membrane loading control. Amounts of plasma membrane-localized GLUT1 and GLUT3 appeared to be similar in both samples. Although levels of the insulin-responsive transporter GLUT4 were higher in membrane preparations from MCF10A cells cultured in the presence of insulin, GLUT4 was readily detectable at the plasma membrane in MCF10HER2 cells maintained long-term in the absence of insulin in serum-free media. (B) MCF10HER2 cells were maintained in serum-free media with out insulin and MCF10A cells were incubated for 18 hours in the absence of insulin in serum-free media, then both cell lines were cultured with or without insulin for 30 minutes. Plasma membrane localized proteins were harvested and probed for GLUT4. In MCF10HER2 cells cultured without insulin basal levels of plasma membrane localized GLUT4 were calculated to be 44% higher than basal GLUT4 plasma membrane levels in MCF10A cells cultured without insulin. In both cell types, insulin induced an increase in GLUT4 at the plasma membrane. The highest levels were observed in the MCF10HER2 cells plus insulin condition. (C) bands in (B) were quantified (optical density) by Quantity One software from Bio-Rad Laboratories. Levels of GLUT4 were normalized to transferrin receptor levels in each context and expressed as a ratio. Relative normalized expression is compared to MCF10A minus-insulin results.

We hypothesized that the relatively high levels of glucose uptake by insulin-independent MCF10HER2 cells was partly due to increased expression of facilitated glucose transporters. Glucose transporters 1 and 3 (GLUT1,GLUT3) are constitutively expressed at the plasma membrane and contribute to basal levels of glucose transport in most cell types, and both transporters have been reported to be transcriptionally up-regulated in cancer cells [26], [27]. In addition, activation of IR signaling by insulin induces translocation of glucose transporter 4 (GLUT4) largely from perinuclear compartments to the plasma membrane in insulin responsive cells [25]. We isolated plasma membrane-localized proteins from MCF10A cells cultured in insulin-containing media and from MCF10HER2 cells cultured in insulin-free media, and immunobloted for GLUT1, GLUT3 and GLUT4. Under these conditions, the amount of GLUT1 and GLUT3 detected in the plasma membrane fraction of both cell types were similar (Figure 3A). Comparing MCF10A and MCF10HER2 plasma membrane GLUT4 levels shown in Figure 3A, higher levels of this insulin-responsive transporter were observed in membrane preparations from MCF10A cells cultured in the presence of insulin, yet levels were readily detected in membrane preparations from MCF10HER2 cells maintained in the absence of insulin. In a follow-up experiment, we compared MCF10A and MCF10HER2 cells cultured with or without insulin and observed that in each condition MCF10HER2 cells had higher plasma membrane GLUT4 levels than MCF10A cells. In MCF10HER2 cells cultured without insulin basal levels of plasma membrane-localized GLUT4 were 44% higher than basal GLUT4 levels detected in the plasma membrane preparations from MCF10A cells cultured without insulin. In both cell types, insulin treatment induced an increase in GLUT4 at the plasma membrane (Figure 3B). These results indicate that insulin induces GLUT4 translocation in both MCF10A and MCF10HER2 cells. Relative to non-induced basal levels observed for MCF10A, there was an increase in non-induced basal GLUT4 localization at the plasma membrane of MCF10HER2 cells, which could partly contribute to their insulin-independent glucose uptake. Notably, the highest levels of plasma membrane-localized GLUT4 were observed in transformed cells exposed to insulin. The relative levels of plasma membrane-localized GLUT4 in MCF10A and MCF10HER2 cells treated with and without insulin shown in Figure 3B correlated with the relative amounts of glucose uptake shown in Figure 2.

### Interrogating dynamic gene expression data to discover a mechanism for the altered metabolic phenotype in HER2 transformed cells

The above results indicate that oncogene-transformed cells acquire the ability to transport relatively high levels of glucose in an insulin-independent manner, and also suggest that GLUT4 may in part play a role in this change. In order to understand the mechanistic basis for this phenotype, we compared the HER2-regulated transcriptome in MCF10A cells with the activated HER2 oncogene-regulated transcriptome in MCF10HER2 cells to identify changes in gene expression that could play a role in HER2 oncogene-driven increased insulin-independence. We treated MCF10A and MCF10HER2 cells with the HER2-specific small molecule kinase inhibitor (CP724,714, 1 µM) to block HER2 kinase activity, and thus HER2-directed gene expression, in both cell types over the course of 45 hours. In that time mRNA was collected every three hours (16 time points) and genome-wide expression was analyzed by microarray. Time series gene expression analysis showed dynamic gene expression level changes that were regulated by HER2 as a function of time in each of the cell lines.

**Figure 4:**
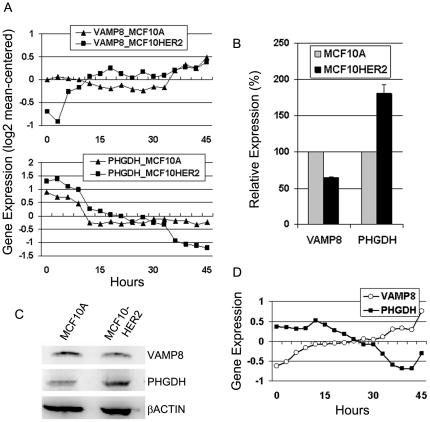
HER2 oncogene-regulated expression of PHGDH and VAMP8. (A) PHGDH and VAMP8 gene expression levels as a function of time after HER2 kinase activity was inhibited by treatment with CP724,714 (1 µM) in MCF10A cells cultured in insulin-containing media and MCF10HER2 cells cultured in insulin-free media. mRNA was collected every three hours for 45 hours total, and measured by microarray. Data are log2 transformed and mean-centered. (B) Steady-state expression levels of VAMP8 in MCF10A and MCF10HER2 cells were compared by real-time RT-PCR (*bars*, standard deviation). (C) Immunoblot analysis of VAMP8 and PHGDH in whole cell lysates from MCF10A and MCF10HER2 cells. (D) VAMP8 and PHGDH expression as a function of time in the HER2-amplified SUM225 breast cancer cell line treated with CP724,714 (1 µM) for 45 hours, these data are log2 transformed and mean centered.

We used differential dynamic gene expression analysis of the time series expression data, described by Shirvani et al. [28] and outlined in Materials and Methods, to discover which genes were differentially regulated by the HER2 oncogene in MCF10HER2 cells compared to HER2 proto-oncogene regulation in the MCF10A cells. We overlaid those genes that were uniquely regulated in MCF10HER2 cells onto canonical pathways for glycolysis and IR signaling (Figures S1 and S2). This analysis resulted in the identification of several differentially regulated genes that function in normal cell metabolism including, ATP citrate lyase (ACLY) and pyruvate dehydrogenase kinase (PDK) that have previously been shown to play a role in cancer cell metabolism [29], [30]. Time-dependent expression for these genes and others regulated by the HER2 oncogene are shown in Figure S3. In addition, we made the novel observations that the HER2 oncogene differentially regulated the expression of phosphoglycerate dehydrogenase (PHGDH) and vesicle-associated membrane protein 8 (VAMP8). PHGDG is an enzyme that commits a glycolytic intermediate to serine biosynthesis [31], and VAMP8 is known to function in the localization of GLUT4 to internal storage vesicles in adipocytes [32], [33]. Figure 4 shows how VAMP8 and PHGDH expression were regulated throughout the time series (Figure 4A). VAMP8 expression was repressed by HER2 signaling in MCF10HER2 cells but not in MCF10A cells. PHGDH expression was more similarly regulated by HER2 in both cell types, however, steady-state levels (0 hours) were nearly 2-fold higher in MCF10HER2 cells compared to MCF10A cells. Furthermore, during the course of the experiment, PHGDH expression changed 5.7-fold in MCF10HER2 cells, but only 2.2-fold in MCF10A cells. The steady-state transcript levels for both genes were verified by real-time RT-PCR (Figure 4B). Also, we performed immunoblot analysis of whole cell lysates, which showed that levels of VAMP8 and PHGDH protein correlated with their gene expression levels (Figure 4C). Finally, results similar to those from MCF10HER2 cells were observed in time-series data obtained from the HER2-amplified SUM225 breast cancer cell line (Figure 4D).

### VAMP8 has a role in regulating GLUT4 localization in MCF10HER2 cells

**Figure 5:**
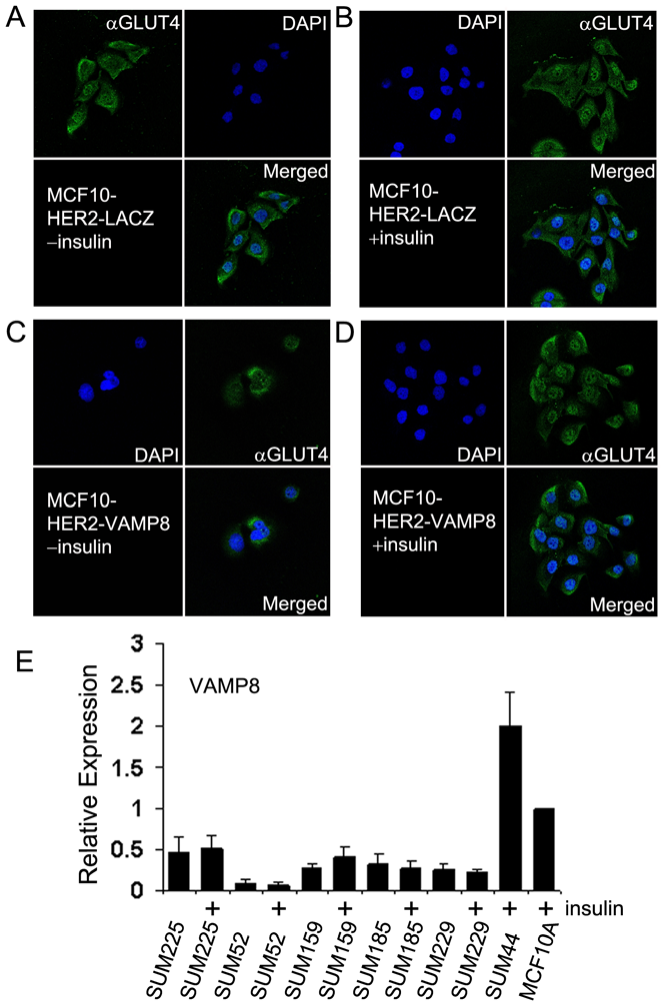
VAMP8 has a role in regulating GLUT4 localization in MCF10HER2 cells. (A and B) MCF10HER2 cells transduced with a control vector expressing LacZ show GLUT4 plasma membrane and intracellular localization with or without insulin in serum-free media. (C) Forced high-level expression of VAMP8 in MCF10HER2 cells causes GLUT4 to be primarily localized to internal stores when insulin is absent from the culture media and (D) the addition of insulin (5 µg/ml) to the culture media shows GLUT4 at the plasma membrane of MCF10HER2 cells over expressing VAMP8. (E) VAMP8 expression levels were low relative to MCF10A cells in 5 breast cancer cell lines, SUM225, SUM52, SUM159, SUM185, and SUM229, which can be maintained without insulin in serum-free conditions. Levels were unchanged when the cell lines were cultured with insulin for 24 hours (*bars*, standard error). Proliferation of the MCF10A line and SUM44 breast cancer cell line requires insulin in serum-free conditions.

VAMP8 is a synaptobrevin that functions in GLUT4 plasma membrane endocytosis [33], [34]. According to microarray, immunoblot and real-time RT-PCR analysis VAMP8 was down-regulated in MCF10HER2 cells compared to MCF10A cells. This suggests that VAMP8 down-regulation contributed to higher steady state levels of GLUT4 on the plasma membrane. We used a lentiviral expression system to infect and over express VAMP8 mRNA and protein in MCF10HER2 cells to test if increased VAMP8 levels would cause GLUT4 to localize to internal storage sites in these cells, and reverse the insulin-independent growth phenotype. When MCF10HER2 cells were transduced to over express VAMP8 we found a dramatic decrease in their growth rate in insulin-free culture conditions, but not insulin-containing conditions. While the cells could still be cultured without insulin, the population doubling time of the VAMP8 expressing cells was increased two-fold. We looked at subcellular localization of GLUT4 in MCF10HER2 cells over expressing VAMP8 and in LacZ expressing control counterparts by indirect immunofluorescence staining and confocal microscopy. In LacZ expressing MCF10HER2 cells, cultured with and without insulin, indirect immunofluorescence detected GLUT4 both intracellularly and at the plasma membrane (Figures 5A and 5B). In MCF10HER2 cells transduced to express high levels of VAMP8 and cultured in insulin-free media, GLUT4 was internally localized and not observed at the plasma membrane (Figure 5C). To observe GLUT4 at the plasma membrane of VAMP8-transduced MCF10HER2 cells required the addition of insulin to the media (Figure 5D). These confocal images provide evidence that VAMP8 has a role in partitioning GLUT4 in breast epithelial cells and just as in adipocytes VAMP8 appears to function in localizing GLUT4 to intracellular storage sights. Forced over expression of VAMP8 in the context of HER2 over expression in MCF10HER2 cells appears to have reinforced intracellular GLUT4 localization in the absence of insulin and hampered insulin-independent proliferation of MCF10HER2 cells.

Next, we examined VAMP8 expression in breast cancer-derived cell lines and found that VAMP8 was expressed at low levels in a panel of breast cancer cells lines compared to expression levels in MCF10A cells (Figure 5E). Most of the breast cancer cell lines we examined were insulin-independent for proliferation; the one exception being the SUM44 cell line, which expressed high VAMP8 levels and also required insulin.

### PHGDH up-regulation by HER2 oncogene signaling has a role in MCF10HER2 insulin-independent proliferation

**Figure 6:**
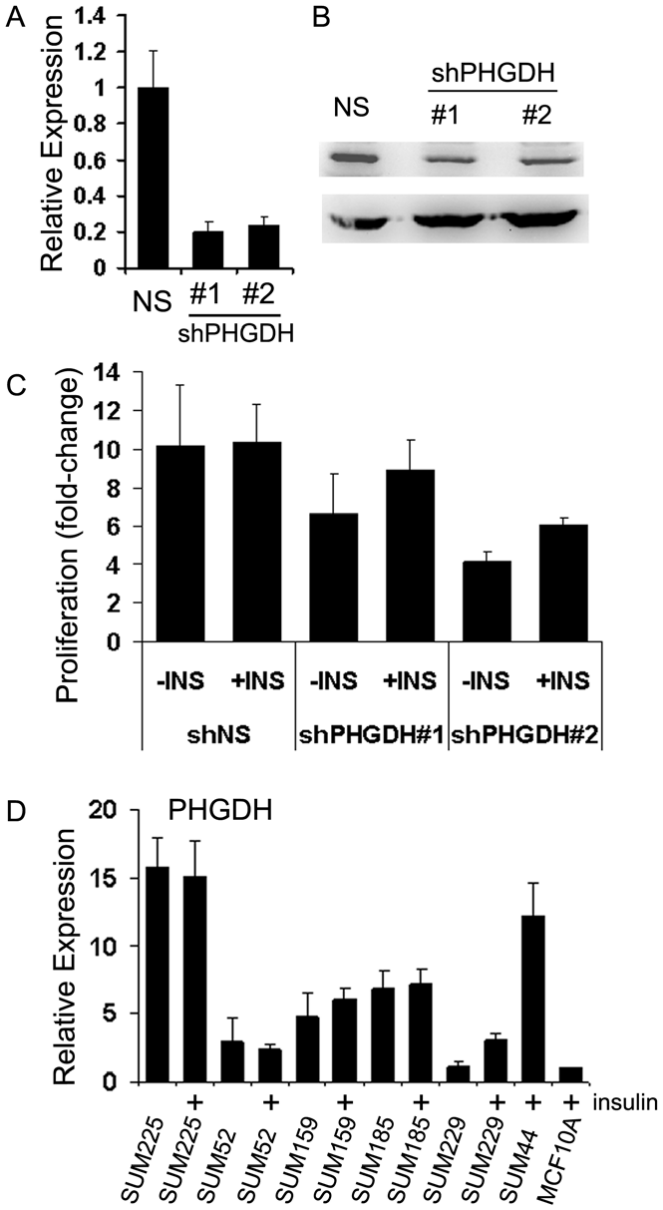
PHGDH upregulation by the HER2 oncogene supports insulin-independent proliferation of MCF10HER2 cells. (A and B) Real-time RT-PCR and immunoblot analysis of pGIPZ shRNA-mediated knockdown of PHGDH mRNA and protein expression (*bars*, standard deviation). The experiment was done using two PHGDH-targeted vectors and a non-silencing control vector. While mRNA levels were reduced nearly 80%, the amount of protein appears to be decreased by only one half. (C) PHGDH knockdown inhibits proliferation of MCF10HER2 cells and the strongest effect, a 60% reduction, was observed for the number 2 construct in minus insulin (-I) culture conditions (*bars*, standard deviation). (D) PHGDH mRNA levels were measured in a panel of breast cancer cell lines relative to MCF10A expression by real-time RT-PCR. SUM225, SUM52, SUM159, SUM185, SUM229 were cultured with and without insulin (5 µg/ml) in serum-free media for 24 hours. PHGDH expression was upregulated in the majority of breast cancer cell lines, including the insulin-dependent SUM44 cancer cell line (*bars,* standard error).

PHGDH catalyzes the rate-limiting reaction that commits an intermediate of the glycolytic pathway to L-serine biosynthesis, which is a substrate for biosynthesis of lipids, proteins and nucleotides [35]. Microarray, immunoblot and real-time RT-PCR data showed that PHGDH was up-regulated in MCF10HER2 cells compared to MCF10A cells. We targeted PHGDH for knockdown in MCF10HER2 cells to investigate if knockdown would inhibit MCF10HER2 cell growth under insulin-free, serum-free conditions. Two individual shRNA constructs effectively knocked down PHGDH mRNA expression levels more than 80%, although protein levels did not reach that level of knockdown (Figure 6A and 6B). Based on the data in Figure 6B showing that the level of protein was approximately reduced by only one half, we conclude that an incomplete knockdown of PHGDH in MCF10HER2 cells attenuated the growth of the cells by as much as 60% compared to control cells transduced by a non-silencing vector. The effect was greater for cells cultured in the absence than in the presence of insulin (Figure 6C). The possible importance of PHGDH up-regulation and L-serine biosynthesis in insulin-independent proliferation of MCF10HER2 cells is underscored by microarray data which showed that three of three enzymes in the pathway of L-serine biosynthesis were also upregulated in proliferating MCF10HER2 cells compared to proliferating MCF10A cells (Figure S4); although phosphoserine phosphatase and phosphoserine aminotransferase were not dynamically regulated by the HER2 oncogene like PHGDH. We examined mRNA from a panel of breast cancer cell lines by real-time RT-PCR and found that compared to MCF10A, PHGDH was expressed at higher levels in the majority, including SUM44 cells, which required insulin for proliferation (Figure 6D).

## Discussion

In the present study, we have shown that in normal human mammary epithelial cells insulin activates the canonical IR pathway to increase glucose uptake, and that oncogene over expression causes insulin-independence of transformed breast epithelial cells, in part by allowing for high level glucose uptake in the absence of insulin. In addition, our results indicate that oncogenes such as HER2 influence the expression of several genes that play a role in glycolysis. Collectively, our results indicate that oncogene-mediated alterations in gene expression play a direct role in metabolic transformation. The insulin-independent phenotype described in this report represents a qualitatively different metabolic condition that promotes tumor cell growth. Furthermore, this study illustrates a novel approach to investigate the oncogene-regulated transcriptome to understand how an oncogene induces altered phenotypes such as metabolic transformation.

It is important to recognize that the insulin-independence phenotype that we have described in this report represents an independence of the metabolic effects of insulin via the IR and not an independence of the mitogenic effects of IGF-I via the IGF-IR. In all of the insulin-independent cells we have studied, there was no evidence of constitutive phosphorylation of the insulin or IGF-I receptors. Furthermore, the IR and IGF-IR substrates IRS1 and IRS2 were not tyrosine phosphorylated in cells proliferating continuously in serum-free and insulin/IGF-I-free media.

Our investigation of insulin-independent growth showed that in addition to oncogenes that function as tyrosine kinases, such as HER2 and FGFR2, functionally unrelated oncogenes like TC1, LSM1 and WHSC1L1 also induce cell transformation to insulin-independent glucose uptake and proliferation. While not all breast cancer cells demonstrate the insulin-independent phenotype, 7 of 9 breast cancer cell lines we examined did. This indicates that insulin-independence is a common in vitro phenotype of oncogene-transformed human breast cancer cells. We also found that oncogene-regulated genes that were implicated in insulin-independence were also commonly altered in their expression in human breast cancer cell lines.

Using isogenic model cell lines to compare nontransformed breast epithelial cells and HER2 over expressing cells we learned that, although the difference might be considered modest by some assessments, non-induced plasma membrane-localized GLUT4 levels were increased in HER2 transformed cells. Moreover, induced plasma membrane levels were decidedly higher in the transformed cells than the induced levels in the nontransformed cells. Evidence to suggest the underlying mechanisms for how the HER2 oncogene caused both increased basal and insulin-induced plasma membrane localization of GLUT4, and the insulin-independent growth phenotype came from our investigation of the transcriptome regulated by the HER2 oncogene. Analysis of time series gene expression data, after inhibiting HER2 activity, led to the discovery of several genes that were differentially regulated by the oncogene in transformed cells, such as ACLY, PDK, INSIG1, and SGK. Notably, we did not find that LDHA was regulated by HER2 in insulin-independent MCF10HER2 cells; however, we did find that LDHA was regulated by HER2 in MCF10HER2/E7 cells that are transformed to a fully growth factor-independent state [36] (see Figure S3), which is consistent with the findings of Zhao et al. [12]. The present data showing that HER2 represses PDK expression does not necessarily contradict reports that show an upregulation of PDK in cancer cells. In cancer cells PDK expression is upregulated by the HIF1α transcription factor [30], [37], and HIF1α is not activated in the MCF10HER2 cells that is by comparison only partly transformed [36], [38]. Considering what was yet unknown in the current literature on cancer cell metabolism we investigated two genes whose expression was HER2 oncogene-regulated, VAMP8 and PHGDH, for their roles in HER2 oncogene-driven insulin-independence. While VAMP8 plays a role in GLUT4 internalization in adipocytes [32], its expression has not previously been reported to be regulated by an oncogene in transformed cells. Furthermore, our data indicate that oncogene-mediated VAMP8 down-regulation in breast epithelial cells may disrupt the balance of mechanisms that partition GLUT4 into internal storage and plasma membrane locales, thus increasing the likelihood that GLUT4 will be plasma membrane localized.

In addition to the HER2 oncogene-dependent down-regulation of VAMP8, we discovered evidence to suggest that the HER2 oncogene-dependent up-regulation of PHGDH expression also functions in promoting insulin-independent proliferation of HER2 transformed cells. PHGDH has recently been recognized to be highly expressed in primary breast tumors [39]. PHGDH up-regulation, which was observed in a majority of the breast cancer cell lines we examined, increases glucose-derived serine production [40]. This relates to an increased utilization of glucose, which could contribute to an increased rate of facilitated glucose transport into transformed cells. Also, increased PHGDH effects increased biosynthesis, which is necessary for increased cell proliferation. These results suggest that the phenotypic effects of oncogene-driven transformation depend on a coordinated change in the expression of many genes.

We recently published a study that more generally explores by computational and systems-level analyses the entire transcriptome that is dynamically regulated specifically by HER2 oncogene signaling [38]. That report concludes that the activated HER2 oncogene directly regulates a transcriptome comprising more than 2000 genes that is quantitatively and qualitatively different from the transcriptome regulated by the proto-oncogene. Further, the HER2 oncogene-regulated transcriptome impacts on many different cellular systems and signaling pathways that affect cancer cell behavior. In the current study, we focus on one phenotype that can be observed in vitro, which distinguishes transformed and cancer cells from nontransformed cells, i.e., the insulin-independent proliferation of oncogene-transformed breast epithelial cells and breast cancer cells in serum-free media. Transcriptomic and pathways-level analyses lead us to further investigate the role of two genes that before now did not have a place in our understanding of the metabolic transformation of breast cancer cells. The results of our pathways-level analysis here reinforce the notion that no single gene can account for a complicated disease such as cancer, moreover, it is unlikely that a single gene can account for a single dysregulated function. Through systems-level approaches we can begin to uncover coordinated networks of genes that underlie transformed phenotypes.

The results of this study are timely considering recent interest in targeting the IR, or co-targeting the IR and IGF-IR, as an approach to cancer therapy. There is strong evidence that insulin promotes tumor cell growth via the IGF-IR and IR [41], [42]. While it is clear that insulin can promote growth of nontransformed breast epithelial cells and breast cancer cell lines, results of our study suggest how oncogene activation can affect gene expression and induce insulin-independent proliferation and glucose uptake in the absence of IR and IGF-IR activity. Since oncogenes regulate so many aspects of the malignant phenotype, including metabolic transformation, targeting activated oncogenes when ever possible in clinical trials using targeted agents is of increasing importance.

## Materials and Methods

### Cell Lines and Cell Culture Conditions

The MCF10A human mammary epithelial cell line [43] was cultured in SFHIE medium (Ham's F-12 medium supplemented with 0.1% bovine serum albumin, fungizone (0.5 µg/ml), gentamicin (5 µg/ml), ethanolamine (5 mmol/L), HEPES (10 mmol/L), transferrin (5 µg/ml), 3,3,′5-Triiodo-_L_-Thyronine (T_3_) (10 µmol/L), selenium (50 µmol/L), hydrocortisone (1 µg/ml), insulin (5 µg/ml or otherwise as indicated in figure legends), and 10 ng/ml epidermal growth factor (EGF)). MCF10HER2 cells, derived from MCF10A cells by stable over expression of HER2 [44], were grown in the same culture media as MCF10A cells but without insulin (SFHE); SUM225, SUM52, SUM159, SUM185, SUM229 [45], [46], [47] and MCF10HER2/E7 [36], all previously developed in our laboratory, were cultured in SFH medium without EGF or insulin. The SUM44 cell line, also previously developed in our laboratory [48], was maintained in SFH with insulin. All cells were cultured at 37°C in a humidified incubator containing 10% CO_2_ and were maintained free of mycoplasma. CP724,714 was used at 1 µM (Pfizer Inc, Groton, CT).

### Whole Cell Lysates and Plasma Membrane Protein Isolation

For whole cell lysate preparation, cells were rinsed twice with ice cold HBSS (Life Technologies, Grand Island, NY) and then lysed on ice with a buffer consisting of Tris-HCl (50 mmol/L, pH 8.5), NaCl (150 mmol/L), 1% NP40 (ICN Biomedical, Inc., Aurora, OH), EDTA (5 mmol/L) supplemented with sodium orthovanadate (5 mmol/L), phenylmethsulfonyl fluoride (50 µg/ml), aprotinin (20 µg/ml), and leupeptin (10 µg/ml). Lysates were spun at 14,000 x *g* at 4°C for 10 minutes and then analyzed for protein using the Bradford method with reagents from Bio-Rad Laboratories (Hercules, CA). Plasma membrane proteins were isolated from sub-confluent plates treated for 1/2 hour with or without insulin using Pierce Cell Surface Protein Isolation Kit, according to manufacturer's instructions (Pierce, Rockford, IL). Briefly, cells were washed twice with PBS then labeled with EZ-Link Sulfo-NHS-SS-Biotin. Cells were subsequently lysed by sonication and labeled proteins were isolated with Immobilized NeutrAvidin-agarose beads. Bound proteins were released by incubating with Laemmli sample buffer.

### Immunoprecipitation and Immunoblotting

Immunoprecipitation and western blotting was performed as previously described [36]. Cells were lysed in a buffer containing 20 mM Tris-HCl (pH 8.0), 137 mM NaCl, 1% NP40, 10% glycerol, 1 mM Na_3_VO_4_, 1 mM phenylmethylsulfonyl fluoride, 1% aprotinin, and 20 µg/ml leupeptin. Protein concentrations were equalized using the Bradford method. For whole cell lysates, Laemmli sample buffer was added and the samples were boiled. For immuno-precipitation, 1 µg of antibody was added to 1 mg of sample and incubated at 4°C for 1 h. Immune complexes were then bound to protein A/G beads for 1 h at 4°C. Immunoprecipitates were washed three times in lysis buffer. Laemmli sample buffer was added and the samples were boiled. IR and IGF-IR immunoprecipitates (50% of eluent), whole cell lysates (100 ug) and isolated cell surface proteins (25 ug) were separated on a 7.5% SDS-polyacrylamide gels and transferred to PVDF membranes by semi-dry electrophoretic transfer with a Bio-Rad Transblot SD unit. Membranes were probed with primary antibodies following supplier's recommendation and secondary peroxidase-conjugated antibodies (anti-mouse or rabbit) from Vector Laboratories (Burlingame, CA). Immunoreactive protein was visualized by enhanced chemiluminescence with reagents from Pierce Laboratories/Thermo Scientific (Rockford, IL). Imaged by a Molecular Imager and Universal Hood II, and Quantity One software from Bio-Rad Laboratories (Hercules, CA). Required primary antibodies: anti-Transferrin Receptor (Zymed Laboratories, South San Francisco, CA), Phospho-tyrosine antibody P-Tyr (BIOMOL, Plymouth Meeting, PA), anti-VAMP (Abcam, Cambridge, MA), anti-PHGDH (Abcam, Cambridge, MA), anti-GLUT1,3,4 (Millipore, Billerica, MA), anti-IRS1 and IRS2 (Millipore, Billerica, MA), anti-IGF-IRβ and anti-IRβ (Santa Cruz Biotech, Santa Cruz, CA).

### Real-time RT-PCR

RNA was extracted from cells using the Qiagen (Valencia, CA) RNeasy kit. RNA was converted into cDNA via a reverse transcription reaction using oligo dt primers and the SuperScript III First-Strand Synthesis System (Invitrogen, Carlsbad, CA). Primer sets specific to approximately 100 bp sequences of target genes and controls (PUM1 and GAPDH) were ordered from Invitrogen. Real-time RT-PCR was done in 25 µL reactions, in 96-well plates, using 100 ng cDNA and the FastStart SYBR Green Master Mix (Roche Diagnostics, Mannheim, Germany). Reactions were done at minimum twice in triplicate using the Bio-rad iQ5 real-time PCR machine (Bio-Rad Laboratories, Hercules, CA). Cycles to threshold values were normalized to values for GAPDH and PUM1 and calibrated to MCF10A cell levels. Control wells containing PCR master mix and primers without sample cDNA emitted no fluorescence after 40 cycles. Relative expression data were calculated as described by Livak and Schmittgen [49]. Primers are available on request.

### Cell Proliferation and Glucose Uptake Assays

The number of nuclei or cells was determined the day after cell plating and at subsequent indicated time points in parallel plated cultures using the Beckman Coulter Z Series systems and protocol (Beckman Coulter, Hialeah, FL). The glucose uptake assay used a technique similar to that described previously [8]. Glucose concentrations in cell culture media were measured enzymatically using the Glucose (GO) Assay kit (Sigma Aldrich, Saint Louis, MO).

### Lentivirus Vectors and Transduction of Cells

The lentiviral expression construct containing the human VAMP8 gene (pLenti6-VAMP8) was established as described earlier [50]. Briefly, the VAMP8 coding sequence was cloned by RT-PCR from the MCF10A cell line using the pENTR/TOPO vector kit (Invitrogen, Carlsbad, CA). Lentiviral expression constructs were created using ViraPower Lentiviral Expression System (Invitrogen, Carlsbad, CA). The construct was sequenced to ensure that the sequence and orientation were correct. Lentivirus was produced by cotransfecting the 293FT cell line with the pLenti6 expression construct and the optimized packaging mix (Invitrogen, Carlsbad, CA). MCF10HER2 cells were transduced with lentivirus and selected with blasticidin. VAMP8 expression levels were detected using real-time RT-PCR and Western blotting. Parallel control infections were done with a LacZ expressing construct, pLenti-LacZ. Lentivirus-mediated shRNA knockdown of PHGDH gene expression was done using the Expression Arrest GIPZ lentiviral shRNAmir system (Thermo Scientific, Huntsville, AL). Lentivirus was produced by transfecting 293FT cells with the combination of the lentiviral expression plasmid DNA targeting PHGDH (catalogue nos. RHS4430-98903591 and RHS4430-99137562) or nonsilencing vector control and trans-lentiviral packaging mix (Thermo Scientific, Huntsville, AL). MCF10HER2 cells were transduced with pGIPZ-derived lentivirus and selected with puromycin. PHGDH expression levels were detected using real-time RT-PCR and Western blotting. Primers are available on request.

### Microarrays and Gene Expression Analysis

Gene expression networks from MCF10A, MCF10HER2 and MCF10HER2/E7 cells were determined from an analysis of global gene expression time series data. Cells were plated so that they reached 75% confluency after 4 days. At this point cultures were treated with the HER-2 kinase inhibitor CP724,714 (1 µM ) and RNA was isolated from parallel plated plates at 0, 3, 6, 9, 12, 15, 18, 21, 24, 27, 30, 33, 36, 39, 42, and 45 hours after addition of inhibitor. Media was changed the day after plating and at the start of treatment. Quantity measurement and the high quality of all mRNA samples were assured by analysis with the NanoDrop 1000, Agilent Bioanalyzer and the Agilent RNA 6000 Nano Kit (Agilent Technologies, Waldbronn, Germany). Expression levels at each time point for each cell and treatment were determined by microarray analyses using the Illumina human Ref8v2 array. Data were processed for quality control and normalized across compared arrays by quantile normalization. MIAME compliant data are accessible through GEO Series accession number GSE23176 (http://www.ncbi.nlm.nih.gov/geo/query/acc.cgi?acc=GSE23176).

2500 genes from MCF10HER2 cells, with 1.7 or greater expression fold-change at any time point in the series, were included in subsequent analysis to identify transcripts that are differentially expressed in MCF10HER2 cells compared to MCF10A. Differential time series gene analysis is previously described [28], MCF10HER2 to MCF10A ratios of gene expression were calculated for each probe at each time point, then normalized to the expression ratio at the zero time point, and finally expressed as the natural logarithm. Genes identified to be differentially expressed had a minimum 2-fold expression ratio at any time point. Ingenuity Systems Path Designer was used to make graphical representations that show the relationships between molecules in the insulin signaling and glycolytic pathways. Molecules are represented as nodes, and a biological relationship between two nodes is represented as an edge (line). All edges are supported by at least 1 reference from the literature, from a textbook, or from canonical information stored in the Ingenuity Pathways Knowledge Base. The intensity of the node color indicates the degree of up (red) or down (green) regulation by the HER2 oncogene.

### Immunohistochemical Analysis

Indirect immunofluorescence analysis was performed on MCF10HER2 cells infected with Lacz or VAMP8 that were plated on glass cover slips, cultured with or without insulin. Two days after plating, cells were washed three times with Tris-buffered saline (TBS), fixed for 3 min in cold 95% methanol, rehydrated by three washes with TBS, and incubated 30 min at 37 C with primary antibody (anti-GLUT4 diluted 1∶1000 in TBS). Bound antibody was detected by staining with a secondary goat anti-rabbit Alexa Fluor 488-conjugated antibody (Invitrogen, Carlsbad, CA) diluted 1∶2000 in TBS, and incubated for 30 min at 37 C. Cover slips were fixed to slides with a drop of ProLong Gold (Invitrogen, Carlsbad, CA) containing DAPI to stain nuclei. Images were recorded using a Leica TCS SP5 Laser Scanning Confocal Microscope (Leica Microsystems, Wetzlar, Germany).

## Supporting Information

Figure S1 **Diagram of the glycolytic pathway showing genes that were differentially regulated by HER2 oncogene signaling in MCF10HER2 cells.** Genes shaded red were upregulated, green were repressed. These genes were differentially regulated compared to HER2 proto-oncogene activity in MCF10A cells.
(TIF)

Figure S2 **Diagram of the insulin receptor signaling pathway showing genes that were differentially regulated by HER2 oncogene signaling in MCF10HER2 cells.** Genes shaded red were positively regulated, and genes shaded green were negatively regulated. These genes were differentially regulated compared to HER2 proto-oncogene activity in MCF10A cells.
(TIF)

Figure S3 **Gene expression levels as a function of time in transformed cells after HER2 kinase activity was inhibited by treatment with CP724,714.** mRNA was collected every three hours for 45 hours total, measured by microarray. Data are log2 transformed, mean-centered. (A) Additional genes that were differentially upregulated by the HER2 oncogene in MCF10HER2 cells. (B) Genes that were differentially down-regulated by HER2 oncogene signaling in MCF10HER2 cells. (C) Notable metabolism genes that were not regulated by HER2 oncogene signaling in MCF10HER2 cells. (D) Genes shown in panel C are regulated by the HER2 oncogene in a transformed cell line that is completely growth factor independent, MCF10HER2/E7.
(TIF)

Figure S4 **Expression levels of L-serine biosynthesis pathway enzymes in MCF10A and MCF10HER2 cells.** Data are the average of three independent microarrays (*Bars,* standard error).
(TIF)
