# Supplementary figures and images for: Oncogene Activation Induces Metabolic Transformation Resulting in Insulin-Independence in Human Breast Cancer Cells
Source: PLoS One. 2011 Mar 17;6(3):e17959. doi: 10.1371/journal.pone.0017959 (PMC3060101; doi:10.1371/journal.pone.0017959)

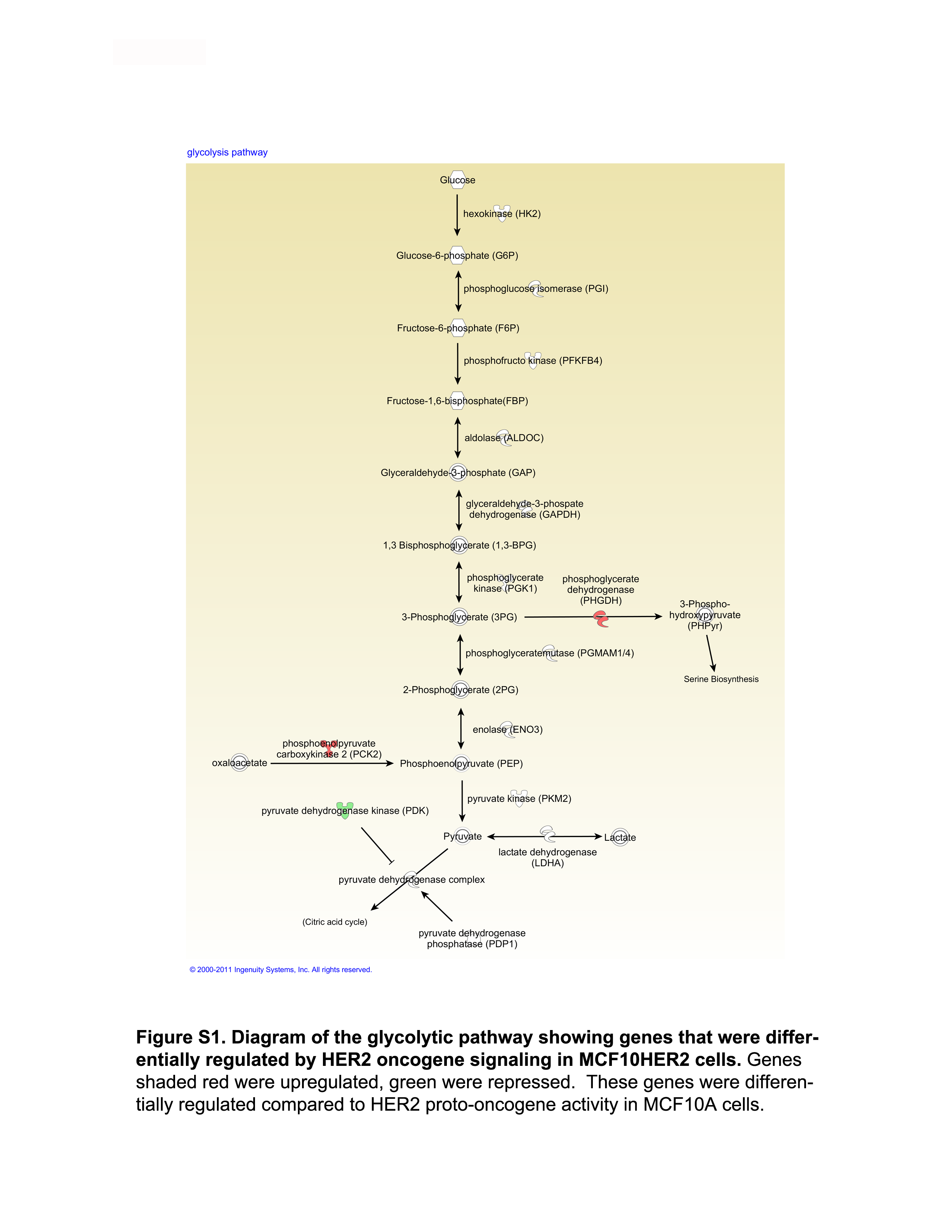

Supplement: Figure S1 — Diagram of the glycolytic pathway showing genes that were differentially regulated by HER2 oncogene signaling in MCF10HER2 cells. Genes shaded red were upregulated, green were repressed. These genes were differentially regulated compared to HER2 proto-oncogene activity in MCF10A cells. (TIF) [file pone.0017959.s001.tif]

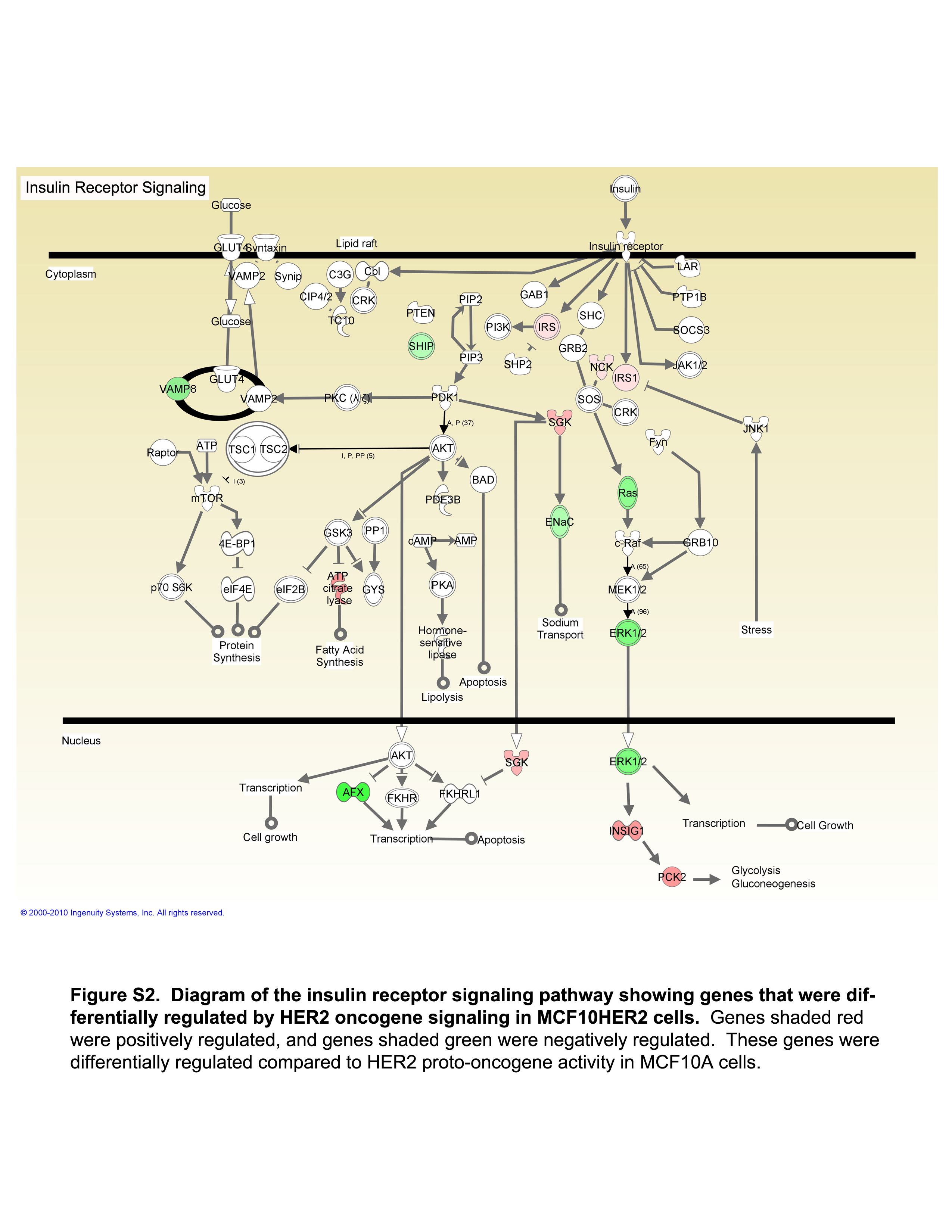

Supplement: Figure S2 — Diagram of the insulin receptor signaling pathway showing genes that were differentially regulated by HER2 oncogene signaling in MCF10HER2 cells. Genes shaded red were positively regulated, and genes shaded green were negatively regulated. These genes were differentially regulated compared to HER2 proto-oncogene activity in MCF10A cells. (TIF) [file pone.0017959.s002.tif]

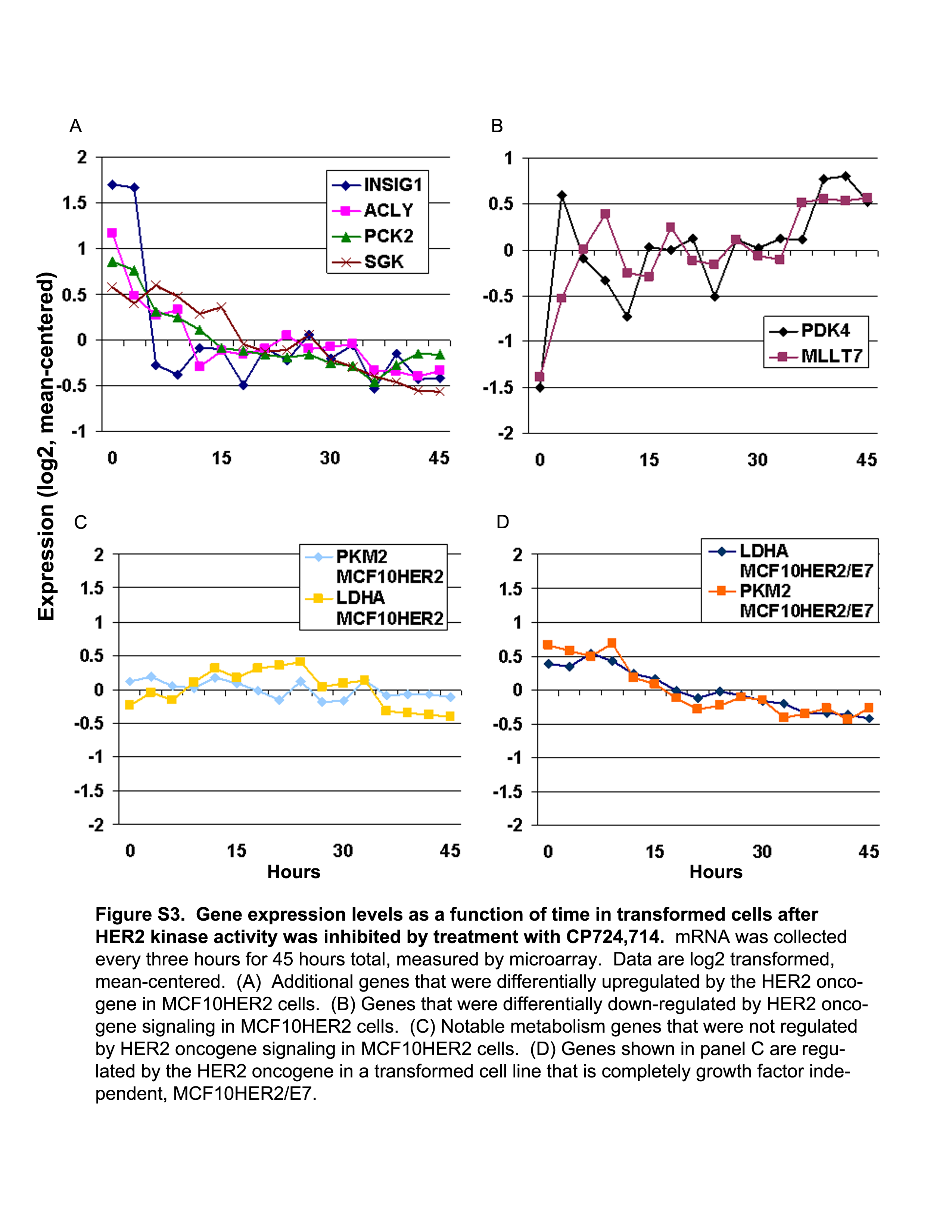

Supplement: Figure S3 — Gene expression levels as a function of time in transformed cells after HER2 kinase activity was inhibited by treatment with CP724,714. mRNA was collected every three hours for 45 hours total, measured by microarray. Data are log2 transformed, mean-centered. (A) Additional genes that were differentially upregulated by the HER2 oncogene in MCF10HER2 cells. (B) Genes that were differentially down-regulated by HER2 oncogene signaling in MCF10HER2 cells. (C) Notable metabolism genes that were not regulated by HER2 oncogene signaling in MCF10HER2 cells. (D) Genes shown in panel C are regulated by the HER2 oncogene in a transformed cell line that is completely growth factor independent, MCF10HER2/E7. (TIF) [file pone.0017959.s003.tif]

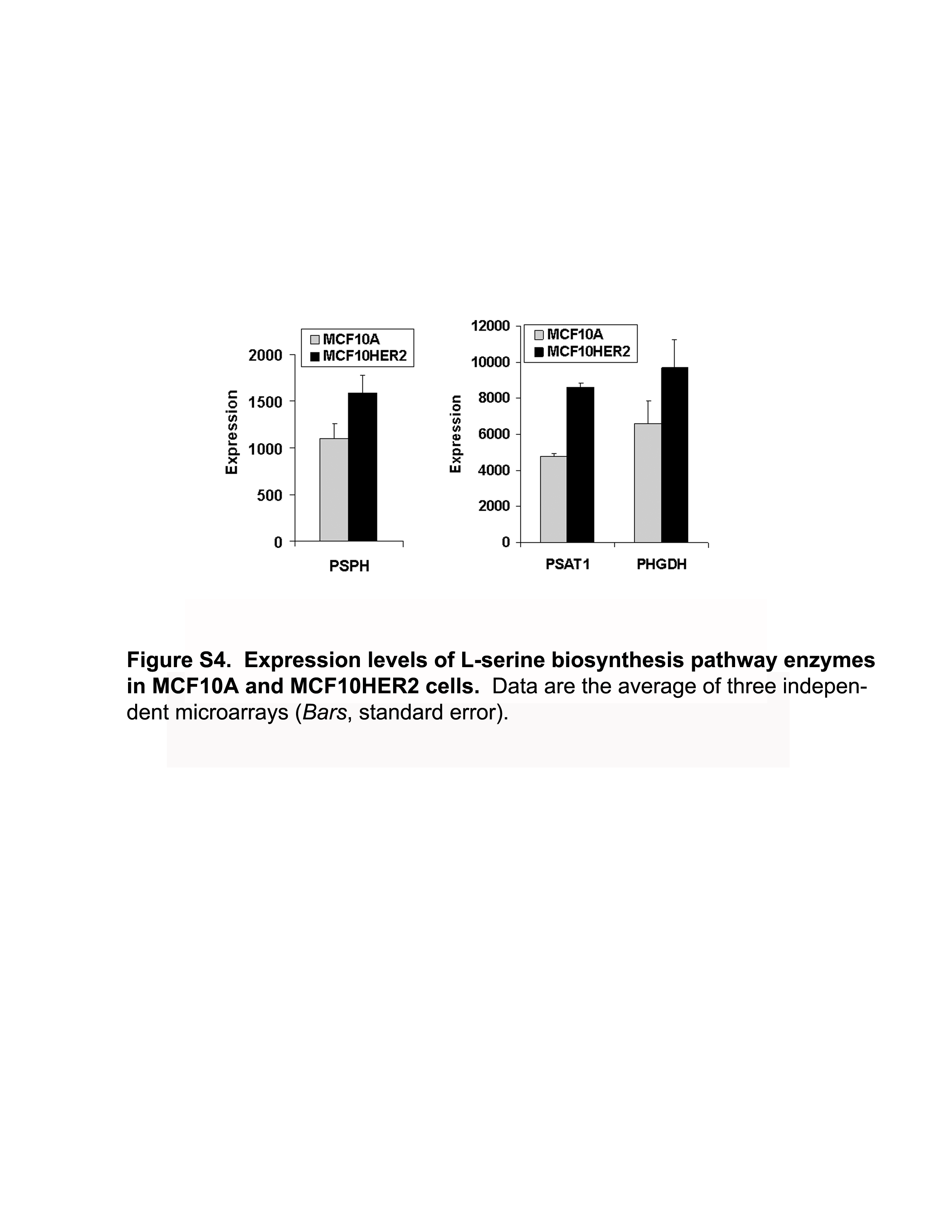

Supplement: Figure S4 — Expression levels of L-serine biosynthesis pathway enzymes in MCF10A and MCF10HER2 cells. Data are the average of three independent microarrays (Bars, standard error). (TIF) [file pone.0017959.s004.tif]
